# 774 Providing Essential Burn Reconstructive Surgery in Resource-Limited Environments Using a Cleft Organization

**DOI:** 10.1093/jbcr/irad045.249

**Published:** 2023-05-15

**Authors:** Zachary Collier, Maria Fernanda Tapia, Atenas Bustamante, Heba Daradkeh, Karel-Bart Celie, William Magee III, Justin Gillenwater, Marzouq Amarin

**Affiliations:** Division of Plastic and Reconstructive Surgery, Keck School of Medicine of USC, Los Angeles, CA, Los Angeles, California; Operation Smile, Bogota, Cundinamarca; Operation Smile, Lima, Lima; Operation Smile, Amman, Amman; Operation Smile, Los Angeles, California; University of Southern California, Los Angeles, California; Keck Medicine of USC, Los Angeles, California; University of Jordan, Amman, Amman, Amman; Division of Plastic and Reconstructive Surgery, Keck School of Medicine of USC, Los Angeles, CA, Los Angeles, California; Operation Smile, Bogota, Cundinamarca; Operation Smile, Lima, Lima; Operation Smile, Amman, Amman; Operation Smile, Los Angeles, California; University of Southern California, Los Angeles, California; Keck Medicine of USC, Los Angeles, California; University of Jordan, Amman, Amman, Amman; Division of Plastic and Reconstructive Surgery, Keck School of Medicine of USC, Los Angeles, CA, Los Angeles, California; Operation Smile, Bogota, Cundinamarca; Operation Smile, Lima, Lima; Operation Smile, Amman, Amman; Operation Smile, Los Angeles, California; University of Southern California, Los Angeles, California; Keck Medicine of USC, Los Angeles, California; University of Jordan, Amman, Amman, Amman; Division of Plastic and Reconstructive Surgery, Keck School of Medicine of USC, Los Angeles, CA, Los Angeles, California; Operation Smile, Bogota, Cundinamarca; Operation Smile, Lima, Lima; Operation Smile, Amman, Amman; Operation Smile, Los Angeles, California; University of Southern California, Los Angeles, California; Keck Medicine of USC, Los Angeles, California; University of Jordan, Amman, Amman, Amman; Division of Plastic and Reconstructive Surgery, Keck School of Medicine of USC, Los Angeles, CA, Los Angeles, California; Operation Smile, Bogota, Cundinamarca; Operation Smile, Lima, Lima; Operation Smile, Amman, Amman; Operation Smile, Los Angeles, California; University of Southern California, Los Angeles, California; Keck Medicine of USC, Los Angeles, California; University of Jordan, Amman, Amman, Amman; Division of Plastic and Reconstructive Surgery, Keck School of Medicine of USC, Los Angeles, CA, Los Angeles, California; Operation Smile, Bogota, Cundinamarca; Operation Smile, Lima, Lima; Operation Smile, Amman, Amman; Operation Smile, Los Angeles, California; University of Southern California, Los Angeles, California; Keck Medicine of USC, Los Angeles, California; University of Jordan, Amman, Amman, Amman; Division of Plastic and Reconstructive Surgery, Keck School of Medicine of USC, Los Angeles, CA, Los Angeles, California; Operation Smile, Bogota, Cundinamarca; Operation Smile, Lima, Lima; Operation Smile, Amman, Amman; Operation Smile, Los Angeles, California; University of Southern California, Los Angeles, California; Keck Medicine of USC, Los Angeles, California; University of Jordan, Amman, Amman, Amman; Division of Plastic and Reconstructive Surgery, Keck School of Medicine of USC, Los Angeles, CA, Los Angeles, California; Operation Smile, Bogota, Cundinamarca; Operation Smile, Lima, Lima; Operation Smile, Amman, Amman; Operation Smile, Los Angeles, California; University of Southern California, Los Angeles, California; Keck Medicine of USC, Los Angeles, California; University of Jordan, Amman, Amman, Amman

## Abstract

**Introduction:**

Cleft non-governmental organizations (NGOs) collaborate with multidisciplinary teams of healthcare professionals to provide safe and effective cleft surgery in resource limited environments. Unfortunately, other significantly disabling conditions like burns, which require similarly multidisciplinary reconstructive interventions, often lack the same degree of local and international support. Therefore, cleft NGOs are well positioned to support essential reconstructive burn care through sustainable service delivery models like those developed for cleft care.

**Methods:**

An NGO with experience delivering global cleft care established a collaborative effort in COUNTRY between local and international burn experts from to provide burn care. Preoperative screening was performed by a local burn surgeon to identify patients. Virtual conferences were held bi-weekly to discuss candidates and operative planning. Following 3 months or preparation, a 5-day surgical program with a mixed team of COUNTRY and international volunteers was held. Patient demographics, interventions, and outcomes were monitored to determine efficacy of this Cleft NGO at delivering essential burn reconstruction.

**Results:**

Of the 52 burn patients who were screened, 32 were surgical candidates, and 20 received a total of 26 surgeries during the 5-day program. Average age was 17 ± 16 years with 65% (13) females and 25% (5) refugees. Most had scald (65%) or flame (30%) burns from household accidents with average age at burn of 8 ± 12 years. Random pattern flaps were used in all cases. Only 1 patient required supplementary skin grafting. Seven patients had multiple operative sites that were addressed with a 2-team approach. No perioperative complications occurred. All patients were followed-up by the in-country team between 7 and 42 days. Five patients presented with minor complications - 5 flaps with partial necrosis and 2 partial dehiscence. None required surgical intervention.

**Conclusions:**

Cleft NGOs have the resources and experience that allows them to implement impactful surgical programs for other conditions that are critically lacking access to essential reconstructive care.

**Applicability of Research to Practice:**

Cleft NGOs can provide safe and effective burn surgery through collaborative local and international efforts aimed at sustainability and capacity building.

